# Dataset of wearable sensors with possibilities for data exchange

**DOI:** 10.1016/j.dib.2019.104978

**Published:** 2019-12-12

**Authors:** Miroslav Muzny, André Henriksen, Alain Giordanengo, Jan Muzik, Astrid Grøttland, Håvard Blixgård, Gunnar Hartvigsen, Eirik Årsand

**Affiliations:** a Spin-Off Company and Research Results Commercialization Center, 1st Faculty of Medicine, Charles University in Prague, Prague, Czech Republic; b Norwegian Centre for E-health Research, Tromsø, Norway; c Department of Community Medicine, UiT The Arctic University of Norway, Tromsø, Norway; d Department of Computer Science, UiT The Arctic University of Norway, Tromsø, Norway

**Keywords:** Wearable device, Health monitoring, Data exchange, Sensors, mHealth, EHR, pHR, Health data aggregators

## Abstract

We performed a search to identify available wearable sensors systems that can collect patient health data and have data sharing capabilities. Findings available in “Wearable sensors with possibilities for data exchange: Analyzing status and needs of different actors in mobile health monitoring systems” [1]. We performed an initial search of the Vandrico wearable database, and supplemented the resulting device list with an internet search. In addition to relevant meta-data (i.e. name, description, manufacturer, web-link, etc.) for each device, we also collected data on 13 attributes related to data exchange. I.e. device type, communication interface, data transfer protocol, smartphone and/or PC integration, direct integration to open health platform, 3rd platform integration with open health platform, support for health care system/middleware connection, recorded health data types, integrated sensors, medical device certification, whether or not the use can access collected data, device developer access, and device availability on the market. In addition, we grouped each device into three groups of actors that these devices are relevant for: electronic health record providers, software developers, and patients. The collected data can be used as an overview of available devices for future researchers with interest in the mobile health (mHealth) area.

**Table:** Specifications Table

Subject	Health Informatics
Specific subject area	Wearable sensors for mobile health monitoring and data exchange
Type of data	Table
How data were acquired	Data was acquired through a search of the Vandrico Wearable database and through an additional web search.
Data format	Raw
Parameters for data collection	Device list was limited to wearable devices capable of collecting any health related data from patients, and which were capable of exchanging that information with other systems.
Description of data collection	Tabulated data file with all available attributes for each device
Data source location	Norwegian Centre for E-health Research, Tromsø, Norway
Data accessibility	Repository name: DataverseNO Data identification number: https://doi.org/10.18710/QXMY88 Direct URL to data: https://dataverse.no/dataset.xhtml?persistentId=doi:10.18710/QXMY88
Related research article	Miroslav Muzny, Andre Henriksen, Alain Giordanengo, Jan Muzik, Astrid Grøttland, Håvard Blixgård, Gunnar Hartvigsen, Eirik Årsand. Wearable Sensors with Possibilities for Data Exchange: Analyzing Status and Needs of Different Actors in Mobile Health Monitoring Systems. International Journal of Medical Informatics, 2019 https://doi.org/10.1016/j.ijmedinf.2019.104017

**Table:** 

**Value of the Data** • These data are useful as a source of known wearable devices with data exchange capabilities, designed to collect various type of personal health data. • Researchers and other actors interested in e-health devices for health data collection can benefit from these data. • These data can potentially be used to generate new ideas for future devices, and for future researchers to more easily get an overview of the current available wearable with these capabilities. • These data adds to the original research article by providing all attributes of each device in a raw form. Other researchers who wants to build on this list can add new devices or attributes, and potentially come up with other groupings and classifications which better serves their needs. • The Vandrico database is no longer available, making this data set unique.

## 1 Data

This dataset contains all information from the 362 wearable devices collected during the Vandrico wearable database search and web search. Devices are grouped into 193 device families, where devices from one manufacturer with similar characteristics are considered one family. Data used in the related research article [1] are stored in a spreadsheet at DataverseNO [2]. There are 20 attributes for each device, 13 attributes related to data exchange capabilities (A-M) and seven attributes of meta-data. Table 1 gives a description of each attribute.

**Table 1:** Wearable devices, attributes not related to data exchange capabilities.

Attribute	Description
Keywords	Custom keywords for assisting in device classifying
Manufacturer	Name of device manufacturer/vendor
Family devices	Numbers of device families identified
System variety	List of devices with similar characteristics found for each manufacturer
URL/System	Link to device manufacturer
Source of information	Link to source used to collect information about device
Short description	Short device description
A) Type of wearable system	Devices are classified into one or more types
B) Communication interfaces	Available integrated communication interfaces
C) Data protocol	Indicates if the devices uses a standardized and open protocol for data transfer, or if a proprietary format is used.
D) Smartphone/PC integration	Indicates to which systems the device can connect
E) Direct integration with health platforms	Indicates if the device can export data directly to existing health platforms
F) 3rd party integration with health platforms	Indicates if and which the device can integrate with a health platform through a 3rd party system.
G) Connection to Health Care System/Middleware	Indicates if device can import data to a health care system or middleware.
H) Health data types	Specifies which data types are available
I) Integrated sensors	Specifies which integrated sensors are available
J) Medical device	Indicates if and which device is approved by the Food and Drug Administration (FDA) or has a Conformité Européenne (CE) marking
K) User data access	Indicates if collected data is accessible to the user
L) Developers access	Indicates if data are available through custom applications, implemented by 3rd party solutions
M) Availability of the device	Indicates the current stats of the device on the consumer marked

## 2 Experimental design, materials, and methods

### 2.1 Search strategy

We performed multiple searches to identify wearable sensors with sharing capabilities. The first search was performed on the Vandrico database web site [3]. We complemented the retrieved device list by performing an additional Google search. For each device in the list, we visited the manufacturer web site and updated device information, as well as identified additional devices by that manufacturer (e.g. newer model). When identifying these additional wearable devices, we followed the same inclusion criteria set by Vandrico (see 4-point list below). Collected attributes are defined in the data chapter (chapter 1).

### 2.2 The Vandrico database

The Vandrico database was the largest online repository of known personal wearable devices. The database was managed by Vandrico Inc. (Canadian, North Vancouver). New devices must satisfy four conditions in order to be accepted into the database. I.e. new devices must be:

1) wearable (i.e. worn on the body throughout its use),
2) controllable (i.e. manageable by the user either actively or passively),
3) enhancing (i.e. device must augment knowledge, facilitate learning or enhance experience), and
4) fully funded (i.e. device must be fully funded, ideally with an availability date and price).

As of May 2018, the database contained 431 devices from 266 companies. An undisclosed buyer acquired the company in 2019, and as of September 2019, the database website was no longer accessible.

## Abbreviations

CE: conformité européenne
CSV: comma separated value
FDA: food and drug administration
